# The relationship of the child’s externalizing and internalizing symptoms with the parent’s maladaptive schemas and attachment style

**DOI:** 10.1192/j.eurpsy.2023.244

**Published:** 2023-07-19

**Authors:** L. Lestyán, B. Szabó

**Affiliations:** ELTE Eötvös Loránd University PPK Institute of Psychology, ELTE Eötvös Loránd University, Budapest, Hungary

## Abstract

**Introduction:**

According to recent studies, there is a relationship between the parent-child attachment style and the child’s externalizing and internalizing problems. However, the parent’s maladaptive schemas were not examined in this relationship before.

**Objectives:**

We aimed to examine the relationship between parents’ maladaptive schemas, well-being, their attachment to their child, and the parent’s perception of the child’s symptoms.

**Methods:**

In our cross-sectional, non-clinical study, 442 parents of children between the ages of 22 and 57 completed the Young Schema Questionnaire (YSQ), Experiences in Close Relationships Questionnaire (ECR), Strength and Difficulties Questionnaire (SDQ) and WHO Well-being Questionnaire (WBI-5). We conducted four mediator analyses. We chose the emotional deprivation schema subscale as the independent variable and attachment anxiety and attachment avoidance subscales as mediators, while the models differed in the dependent variables. We chose the SDQ externalizing symptoms, internalizing symptoms, prosociality subscale, and WHO well-being questionnaire as dependent variables.

**Results:**

The mediation analyses proved to be significant. Emotional deprivation had significant effect on the parent’s attachment anxiety (a_1_ = 0.01, p < .001), avoidance (a_2_ = 0.32, p = .002) and on the parent’s well-being (c_1_ = -0.12 , p < .001), while emotional deprivation was not associated with externalizing symptoms (c_2_ = 0.002, p = .25), internalizing symptoms (c_3_ = 0.001, p = .53) and prosociality (c_4_ = 0.01, p = .26). However, the indirect effects of emotional deprivation through attachment anxiety were significant in case of internalizing symptoms (*a_1_b_5_* = 0.03 [0.02 – 0.05), prosociality (*a_1_b_8_* = -0.004 [-0.01 – -0.0002]) and well-being (*a_1_b_1_* = -0.017 [-0.03 – -0.005]). Furthermore, the indirect effects of emotional deprivation through attachment avoidance were significant in case of internalizing symptoms (*a_2_b_6_* = 0.009 [0.002 – 0.02]) and prosociality (*a_2_b_8_* = -0.007 [-0.01 – -0.0002]). While the indirect effects of emotional deprivation through attachment anxiety (*a_1_b_3_* = 0.007 [-0.001– 0,02]) and avoidance (*a_2_b_4_* = 0.0001 [-0.006 – 0.006]) on externalizing symptoms were not significant. The indirect effects of emotional deprivation through attachment avoidance on the parent’s well-being were not significant (*a_2_b_2_* = -0.002 [-0.01 – 0.006]).

**Conclusions:**

Our results - taking into account the limitations - suggest that there is a relationship between the parent’s emotional deprivation schema and the child’s internalizing symptoms, prosociality, and the parent’s well-being through the parent-child attachment pattern. Attachment style and maladaptive schema have no effect on externalizing symptoms. These results may also have practical implications.

**Disclosure of Interest:**

None Declared

